# Supplementary figures and images for: A brief, theory-driven patient education video reduces high-risk over-the-counter nonsteroidal anti-inflammatory drug (NSAID) use
Source: PLoS One. 2025 Nov 10;20(11):e0323582. doi: 10.1371/journal.pone.0323582 (PMC12599932; doi:10.1371/journal.pone.0323582)

**S1 File: Screening Survey**


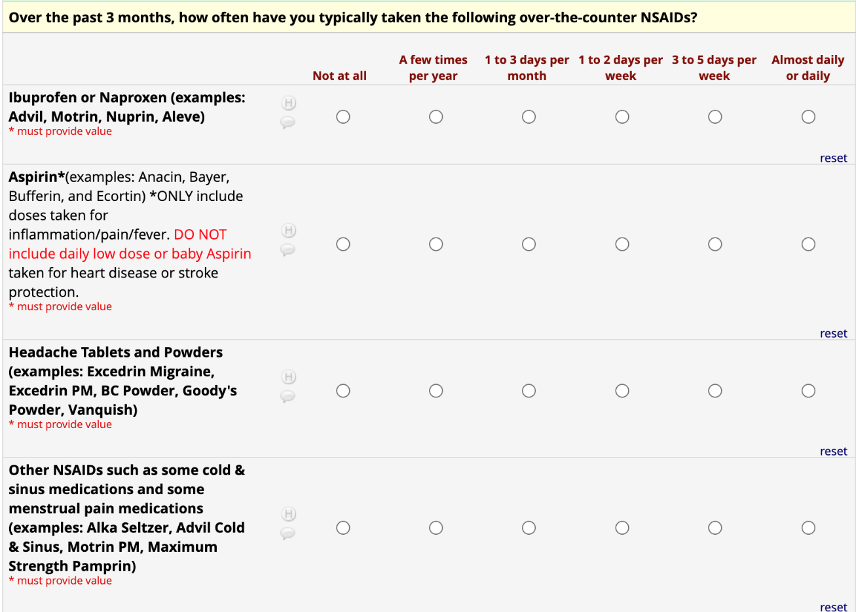

Supplement: S1 File — (DOCX) [file pone.0323582.s001.docx]

**S5 File: Control/Label**


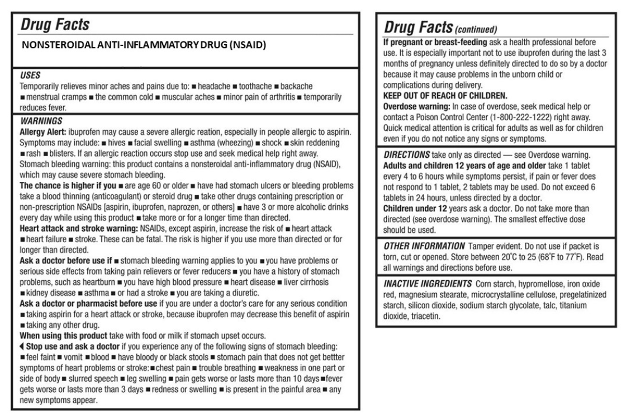

Supplement: S5 File — (DOCX) [file pone.0323582.s005.docx]
