# Supplementary material for: A brief, theory-driven patient education video reduces high-risk over-the-counter nonsteroidal anti-inflammatory drug (NSAID) use
Source: PLoS One. 2025 Nov 10;20(11):e0323582. doi: 10.1371/journal.pone.0323582 (PMC12599932; doi:10.1371/journal.pone.0323582)
Supplement: S2 File — (DOCX) [file pone.0323582.s002.docx]

**S2 File:** **ICD-10 codes used to identify prospective participants**

| **CKD** | **HF** | **HTN** |
| --- | --- | --- |
| I12 | I11.0 | I10 |
| I12.0 | I13.0 | I11 |
| I12.9 | I13.2 | I11.0 |
| I13 | I50 | I11.9 |
| I13.0 | I50.1 | I12 |
| I13.1 | I50.2 | I12.0 |
| I13.10 | I50.20 | I12.9 |
| I13.11 | I50.22 | I13 |
| I13.2 | I50.3 | I13.0 |
| N18 | I50.30 | I13.1 |
| N18.1 | I50.32 | I13.10 |
| N18.2 | I50.4 | I13.11 |
| N18.3 | I50.40 | I13.2 |
| N18.30 | I50.42 | I15 |
| N18.31 | I50.8 | I15.0 |
| N18.32 | I50.82 | I15.1 |
| N18.4 | I50.84 | I15.2 |
| N18.5 | I50.89 | I15.8 |
| N18.6 | I50.9 | I15.9 |
| N18.9 |  |  |
| N19 |  |  |
