# Supplementary material for: A brief, theory-driven patient education video reduces high-risk over-the-counter nonsteroidal anti-inflammatory drug (NSAID) use
Source: PLoS One. 2025 Nov 10;20(11):e0323582. doi: 10.1371/journal.pone.0323582 (PMC12599932; doi:10.1371/journal.pone.0323582)
Supplement: S3 File — (DOCX) [file pone.0323582.s003.docx]

**S3 File: Randomization process**

The randomization process, automated through REDCap, was based on participants' responses to the pre-intervention survey. Using the datetime stamp of each response, participants were assigned to either the VIDEO or CONTROL group. The randomization algorithm aimed to create a nearly even distribution between the groups by utilizing the time difference between the survey start time and the response datetime. Specifically, the calculation involved rounding this time difference to a certain precision to determine the random assignment. Participants were assigned to one group if the rounded result met a specified condition. This approach ensured a systematic and unbiased allocation of participants. The exact calculation used for the study cohort field was:

`if(round(((datediff([survey_start_time],[datetime_stamp],"s"))/2),1)=round(((datediff([survey_start_time],[datetime_stamp],"s"))/2),0),0,
