# Supplementary material for: A brief, theory-driven patient education video reduces high-risk over-the-counter nonsteroidal anti-inflammatory drug (NSAID) use
Source: PLoS One. 2025 Nov 10;20(11):e0323582. doi: 10.1371/journal.pone.0323582 (PMC12599932; doi:10.1371/journal.pone.0323582)
Supplement: S4 File — The video and related images are property of the Virginia Tech Carilion School of Medicine. They are reprinted and made available in this publication under a CC BY license, with permission from the Virginia Tech Carilion School of Medicine, original copyright 2024. (DOCX) [file pone.0323582.s004.docx]

**S4 File: Video**

<https://www.youtube.com/watch?v=zsiJjXk162E>


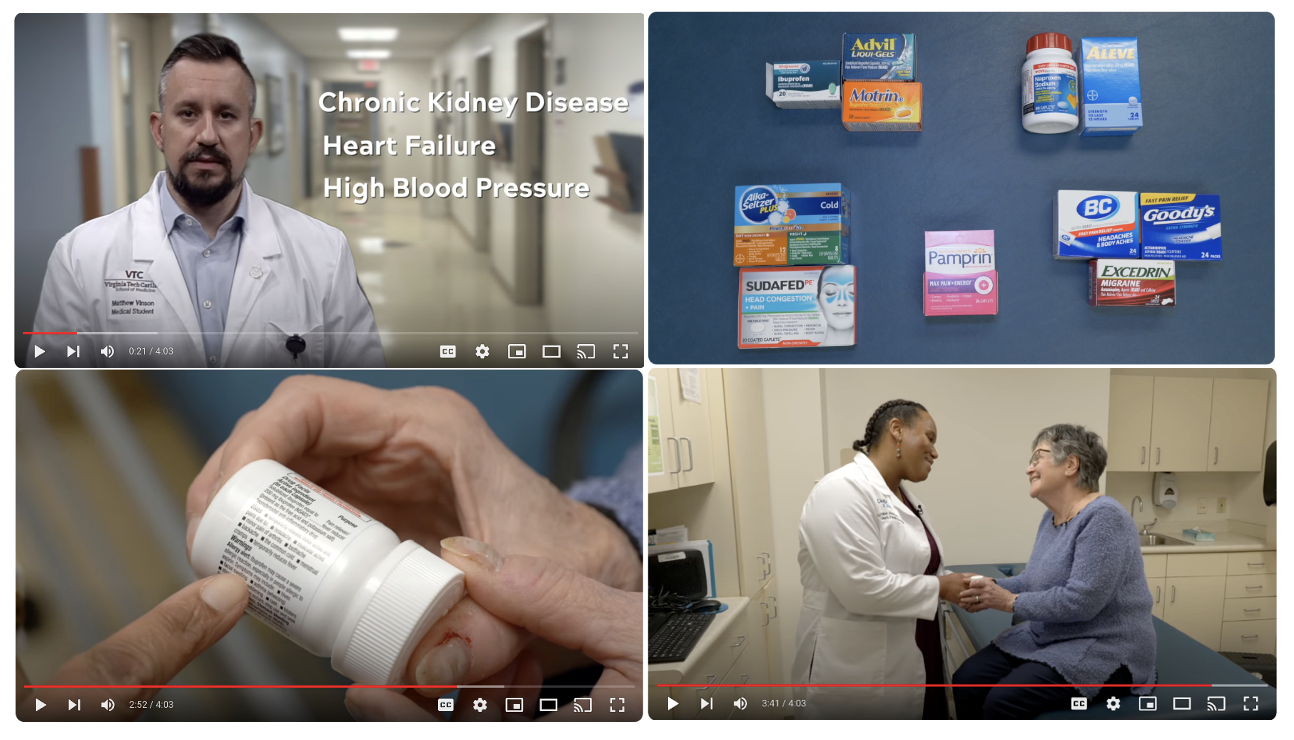


The video and related images are property of the Virginia Tech Carilion School of Medicine. They are reprinted and made available in this publication under a CC BY license, with permission from the Virginia Tech Carilion School of Medicine, original copyright 2024.
