# Supplementary material for: A brief, theory-driven patient education video reduces high-risk over-the-counter nonsteroidal anti-inflammatory drug (NSAID) use
Source: PLoS One. 2025 Nov 10;20(11):e0323582. doi: 10.1371/journal.pone.0323582 (PMC12599932; doi:10.1371/journal.pone.0323582)

**S6 File: Full survey questions**

**Pre-intervention assessment**


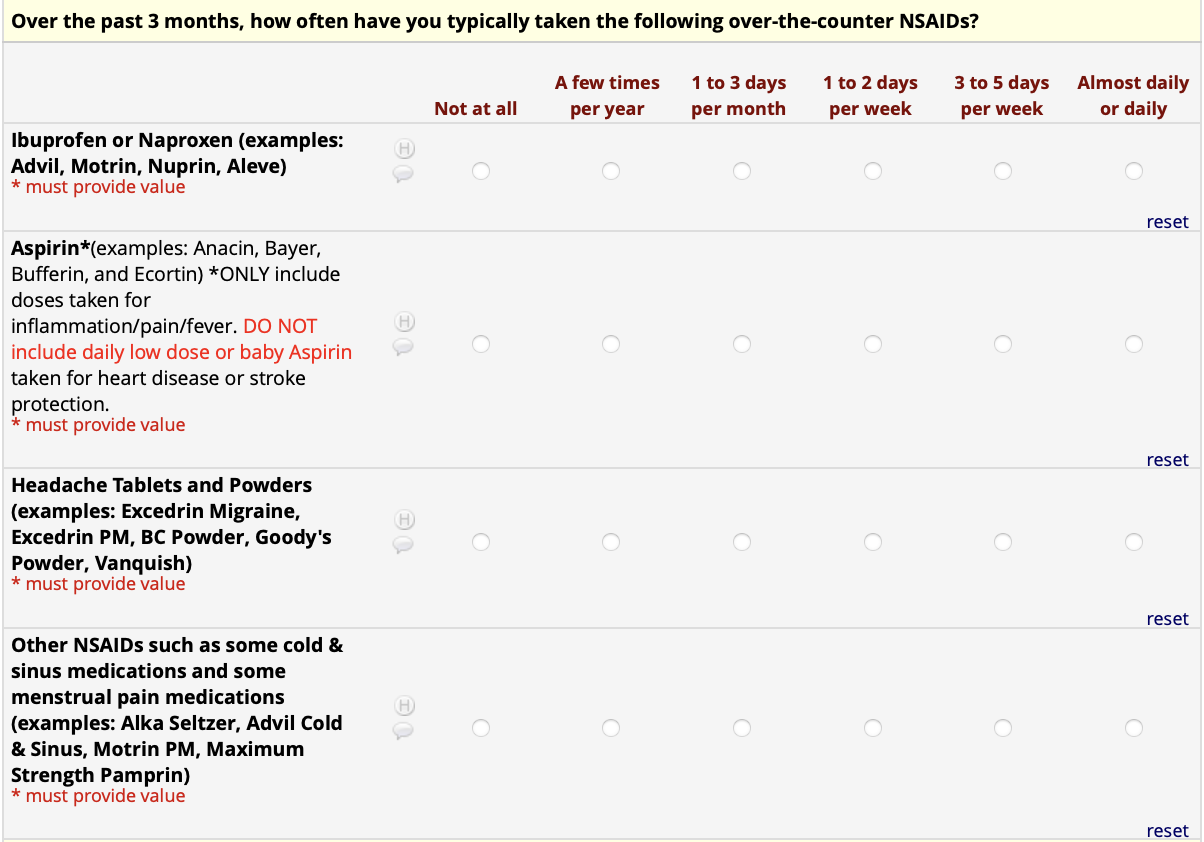


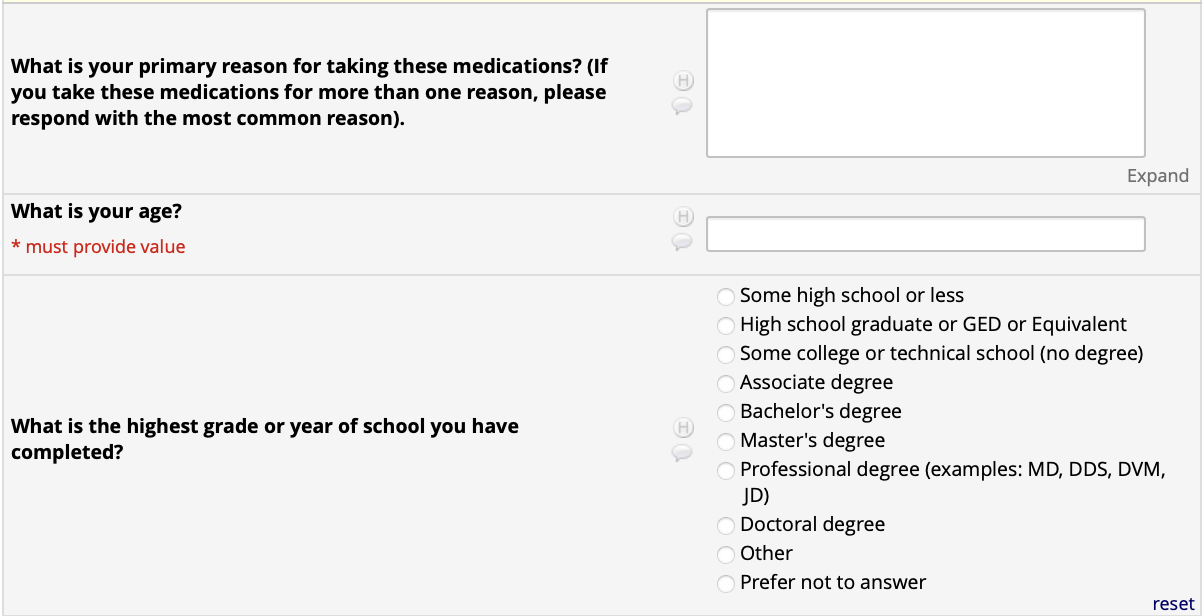


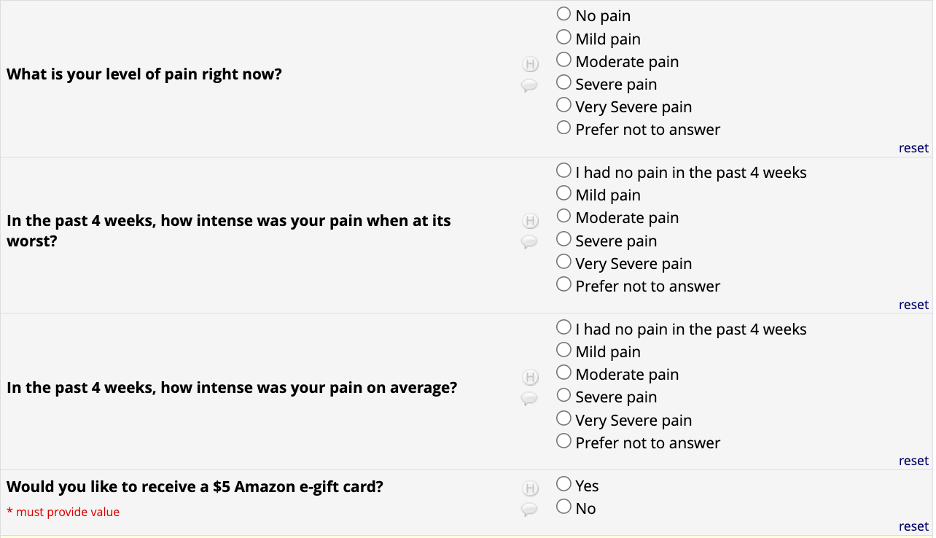


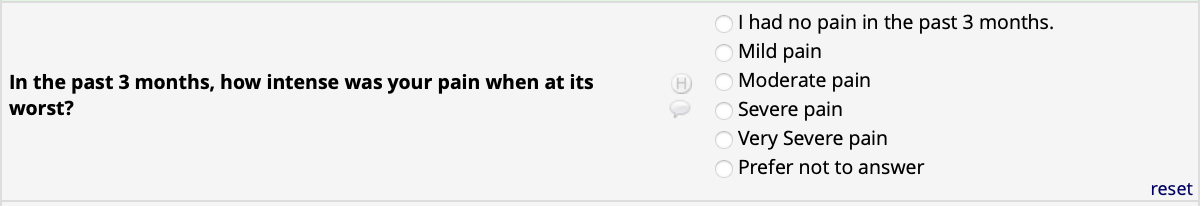


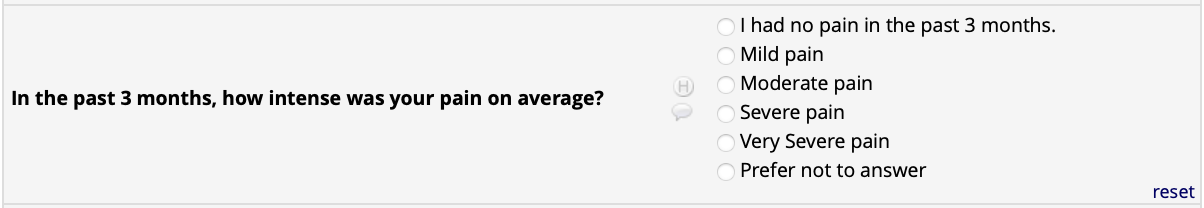


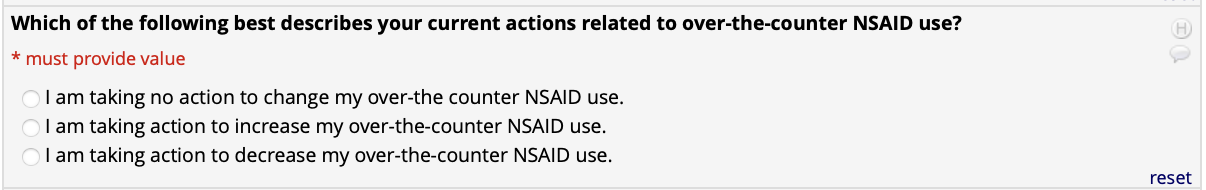


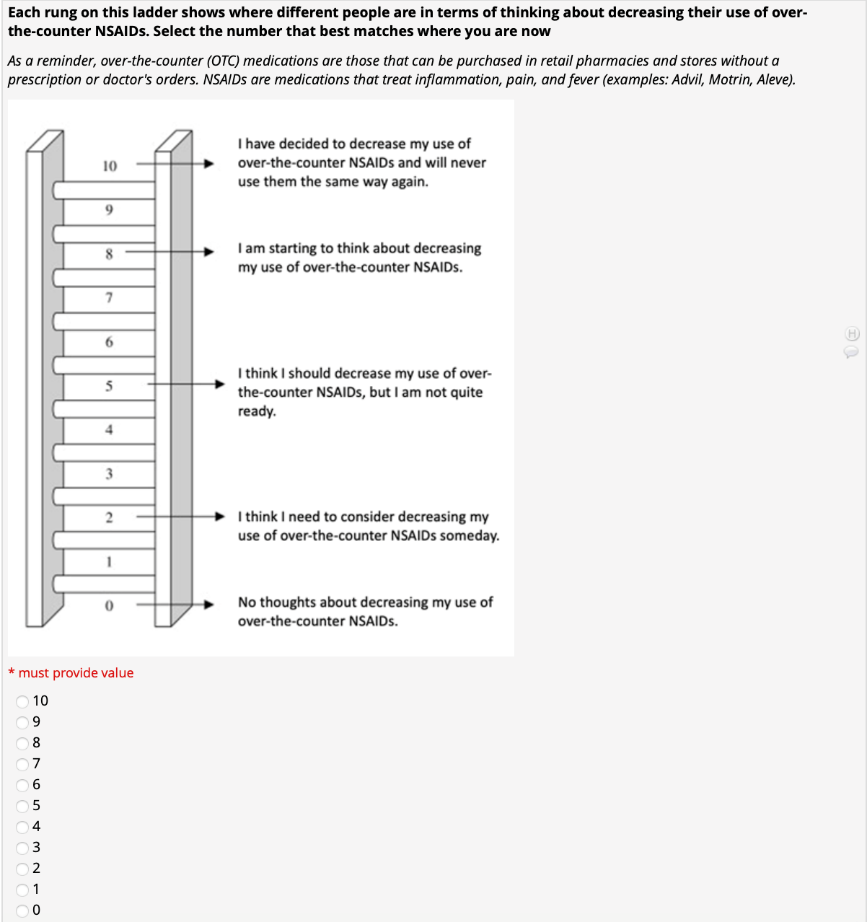


**Post-intervention assessment**


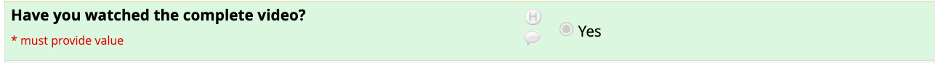
Or


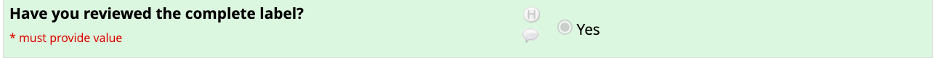


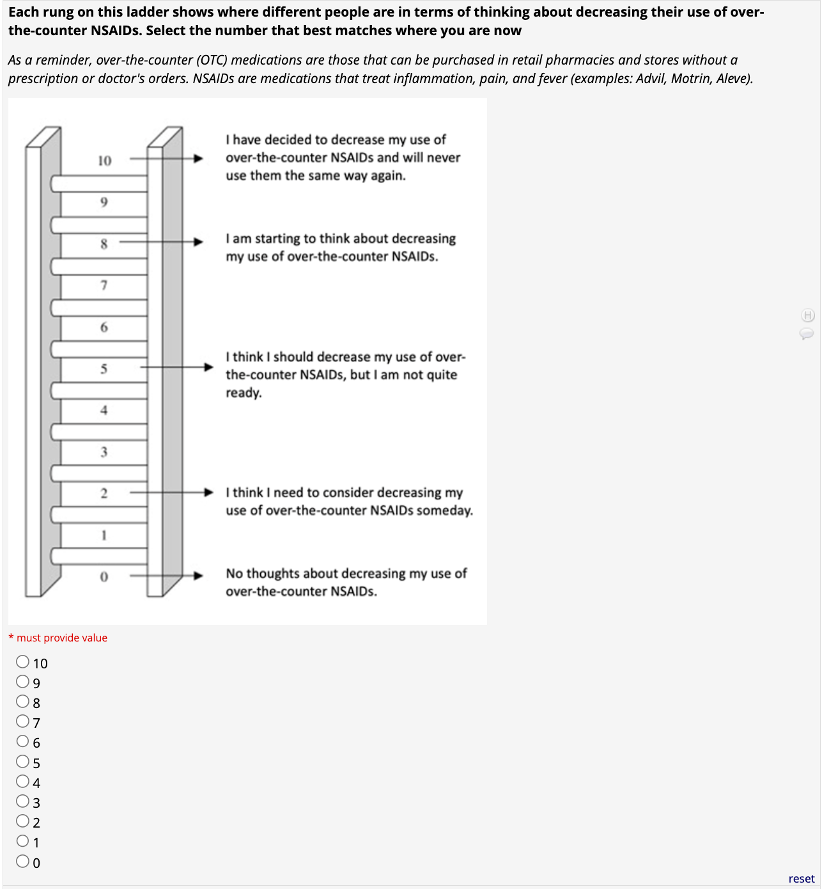


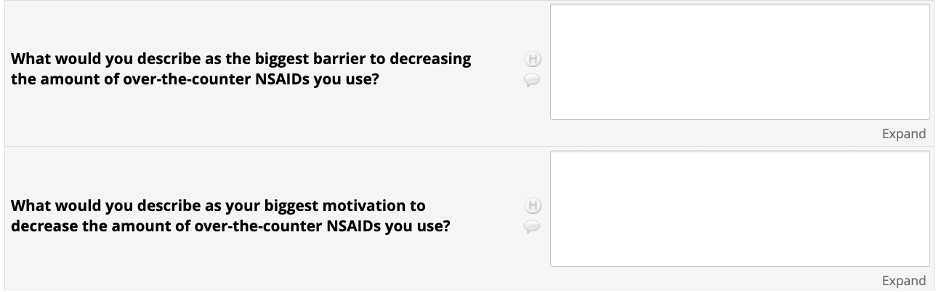


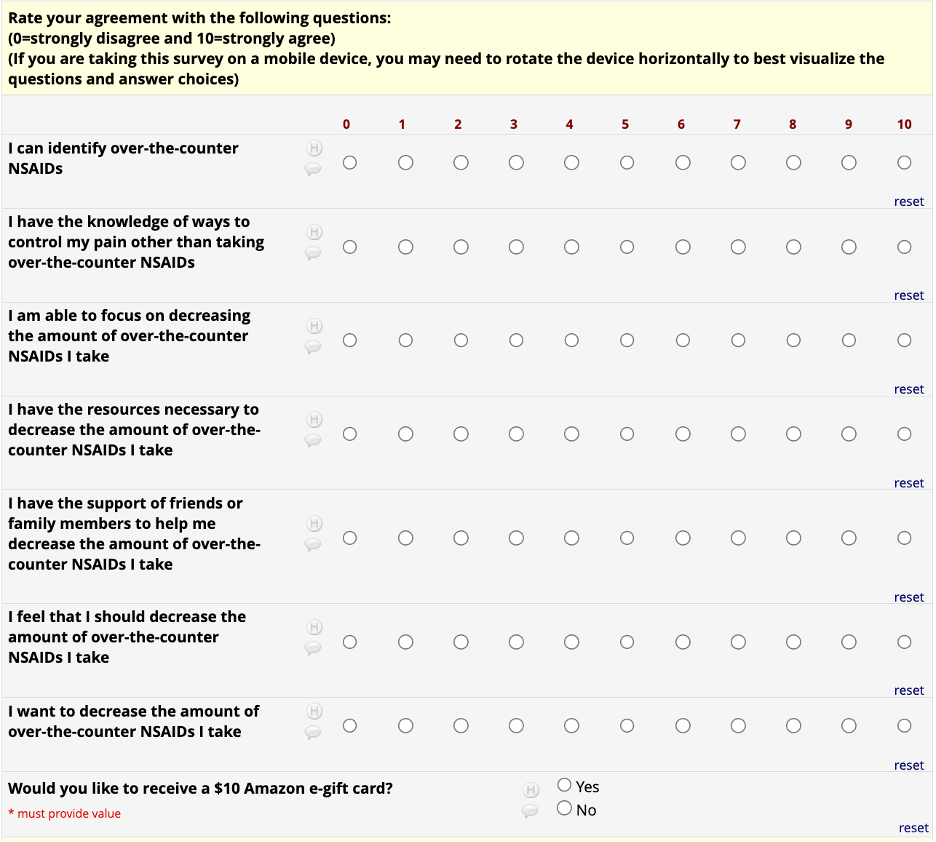


Questions 1-2 = Capability

Questions 3-5 = Opportunity

Questions 6-7 = Motivation

**4-week follow up assessment**


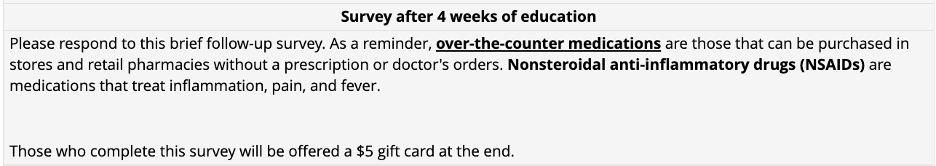


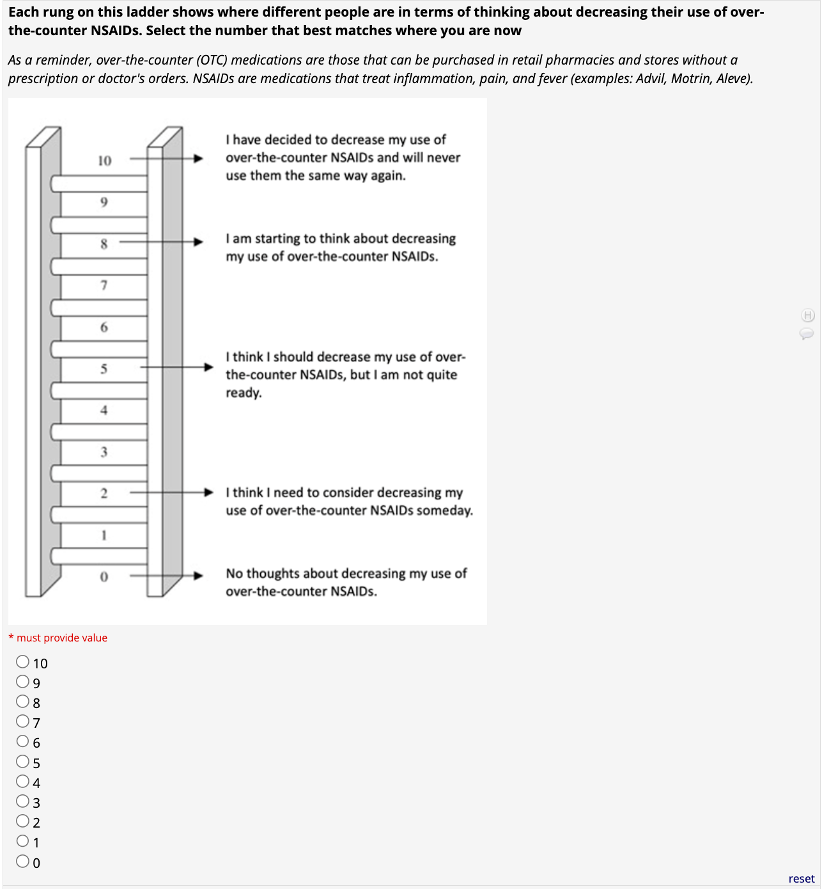


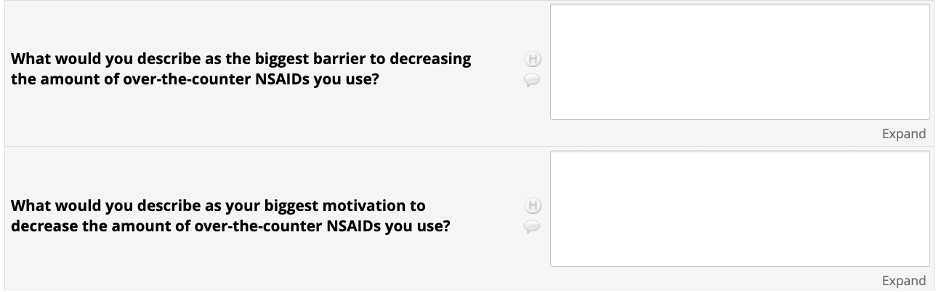


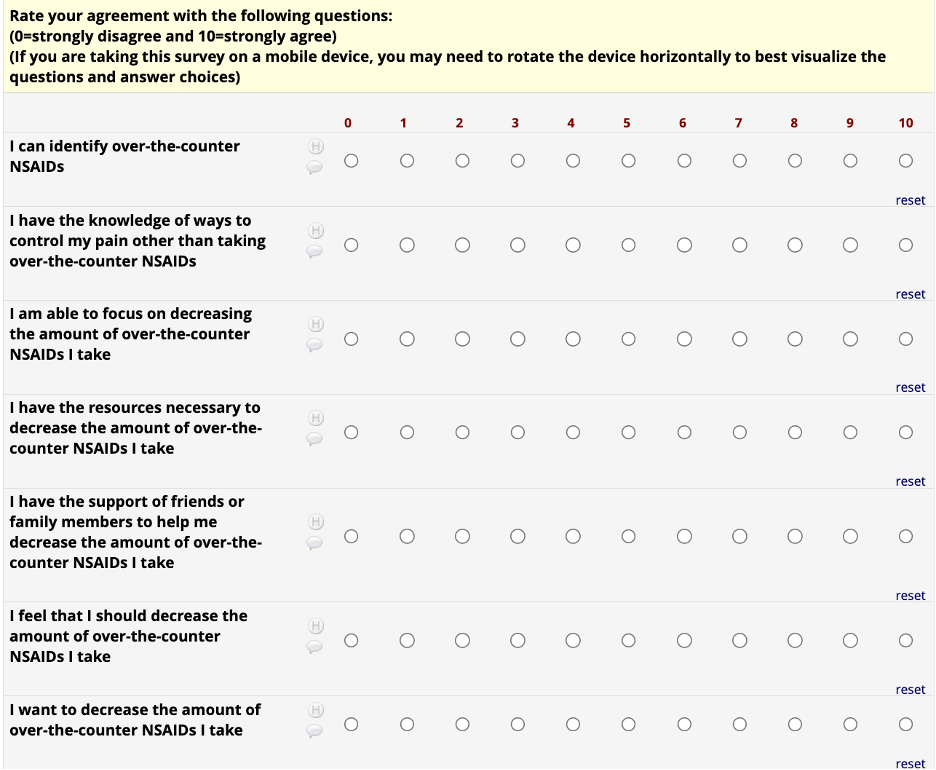


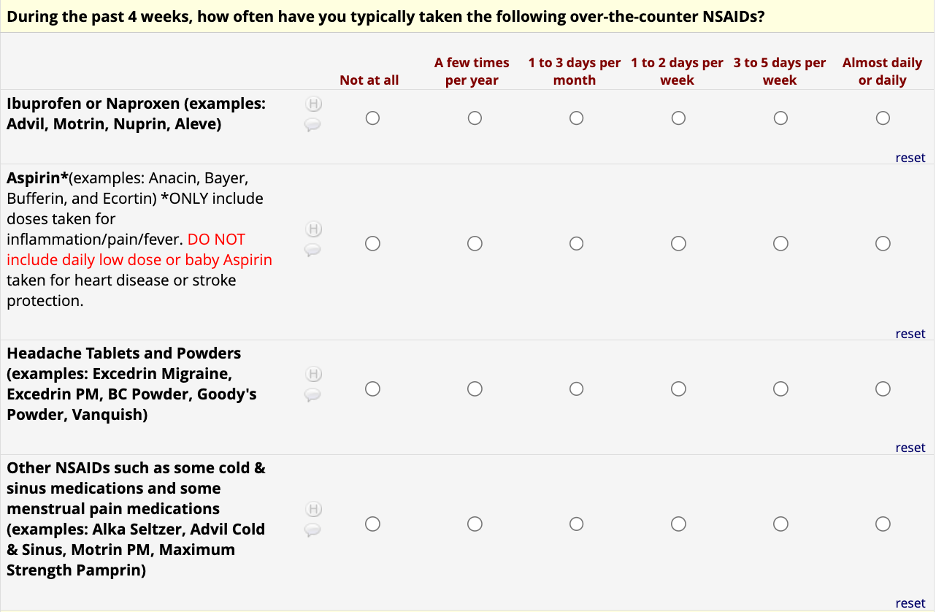


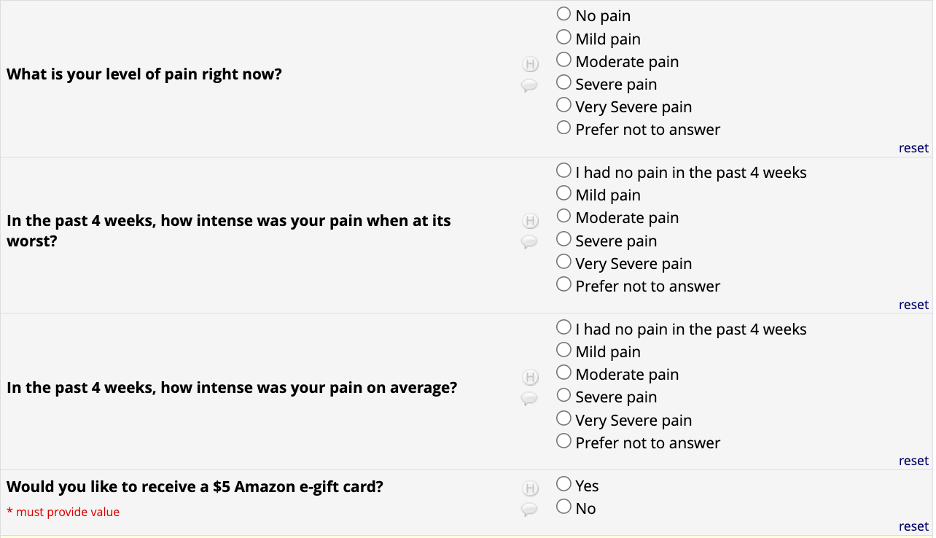

Supplement: S6 File — (DOCX) [file pone.0323582.s006.docx]
