# Supplementary material for: A brief, theory-driven patient education video reduces high-risk over-the-counter nonsteroidal anti-inflammatory drug (NSAID) use
Source: PLoS One. 2025 Nov 10;20(11):e0323582. doi: 10.1371/journal.pone.0323582 (PMC12599932; doi:10.1371/journal.pone.0323582)
Supplement: S7 File — *OTC= over-the-counter, †NSAID = nonsteroidal anti-inflammatory drug, ‡Excluded: 132 participants were excluded due to: (a) reported low-dose aspirin use for cardiovascular disease prevention rather than pain management (n = 63); (b) misclassified as eligible to participate due to administrative error (n = 66); and (c) duplicate responses (n = 3). (DOCX) [file pone.0323582.s007.docx]

**S7 File: CONSORT diagram**


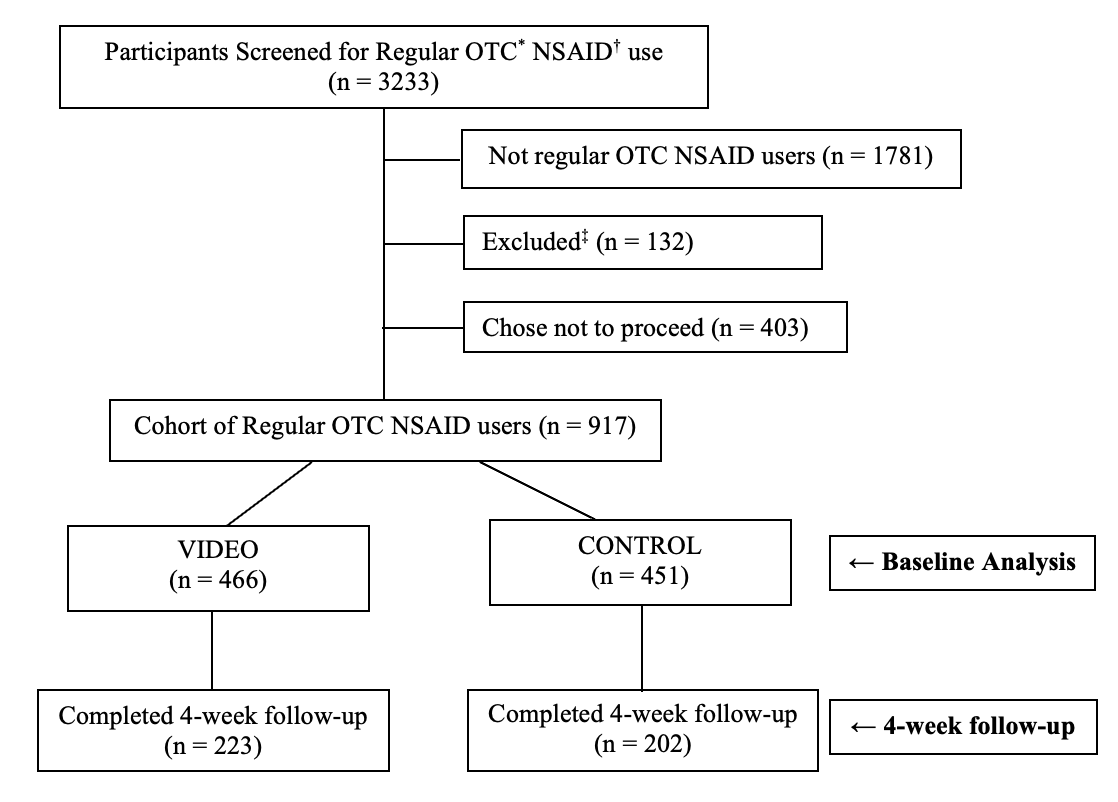


^*^OTC= over-the-counter

^†^NSAID= nonsteroidal anti-inflammatory drug

^‡^Excluded: 132 participants were excluded due to: (a) reported low-dose aspirin use for cardiovascular disease prevention rather than pain management (n=63); (b) misclassified as eligible to participate due to administrative error (n= 66); and (c) duplicate responses (n= 3).
