# Supplementary material for: A brief, theory-driven patient education video reduces high-risk over-the-counter nonsteroidal anti-inflammatory drug (NSAID) use
Source: PLoS One. 2025 Nov 10;20(11):e0323582. doi: 10.1371/journal.pone.0323582 (PMC12599932; doi:10.1371/journal.pone.0323582)
Supplement: Attachment — Submitted filename: Comments to plos one.pdf [file pone.0323582.s008.pdf]

Thank you for your invitation to revise the article entitled:

A brief, theory-driven patient education video reduces high-risk over-the-counter nonsteroidal anti-inflammatory drug (NSAID) use.

I have few minor comments:

1- We need reference in line 85
2- I suggest to add (in patients with CKD, HF, and/or HTN) in the last of the title to be; A brief, theory-driven patient education video reduces high-risk over-the-counter nonsteroidal anti-inflammatory drug (NSAID) use in patients with CKD, HF, and/or HTN.
3- In discussion chapter line 351; we need a reference.
4- Line 254; "In assessing the change in intent from pre- to post-intervention,….." where is the same assessment for control group?
5- In line 299; the authors say "After 4 weeks, intent to decrease use of OTC NSAIDs was similar in VIDEO and CONTROL" …how come while there wase a significant difference in intent between the two groups immediately after intervention (you say), the change in intent was not significantly different between groups after 4 weeks (you say) and there was no significant difference between pre and post 4 weeks period in both groups (you say)? It does make sense if there was a significant difference in intent between the two groups after two weeks also.
6- In conclusion part the authors didn't mention the comparison between the two studied groups as I think overall differences wasn't found. The control group as the authors say "There was also no significant difference between

VIDEO and CONTROL in NSAID Exposure at the 4-week Follow-Up assessment (19.67 (SD: 19.60) and 18.42 (SD: 15.72) dose-days, respectively; p = 0.48).” and also say “From pre-intervention to 4 weeks post-intervention, OTC NSAID Exposure decreased by 9.61 (SD: 14.26) dose-days in VIDEO (a 32.8% decrease) and 10.58 (SD: 16.10) dose-days in CONTROL (a 36.5% decrease), with no significant difference between groups (p = 0.46)”. this means (image of the Food and Drug Administration (FDA) Drug Facts label for OTC NSAIDs) that digested by control group is as effective as video (in the test group) in reducing NSAIDs exposure after the 4 weeks period of the study. The authors frankly mentioned that throughout the context of the article but, they should clarify that in the conclusion part.

7- Overall the article is good and concised.
