# Supplementary material for: A brief, theory-driven patient education video reduces high-risk over-the-counter nonsteroidal anti-inflammatory drug (NSAID) use
Source: PLoS One. 2025 Nov 10;20(11):e0323582. doi: 10.1371/journal.pone.0323582 (PMC12599932; doi:10.1371/journal.pone.0323582)
Supplement: Attachment — Submitted filename: Response to Reviewers_VinsonOTCNSAIDVideo_9.7.25.docx [file pone.0323582.s010.docx]

**Response to Editor**

Journal Requirements:

When submitting your revision, we need you to address these additional requirements.

1. Please ensure that your manuscript meets PLOS ONE's style requirements, including those for file naming. The PLOS ONE style templates can be found at

<https://journals.plos.org/plosone/s/file?id=wjVg/PLOSOne_formatting_sample_main_body.pdf> and

<https://journals.plos.org/plosone/s/file?id=ba62/PLOSOne_formatting_sample_title_authors_affiliations.pdf>

►Our resubmission has addressed PLOS ONE style requirements, including within the title page, manuscript, and for file naming.

2. Thank you for stating the following financial disclosure:

“This research was funded, in part, by a collaborative grant from the Virginia Tech Libraries.”

Please state what role the funders took in the study.  If the funders had no role, please state: "The funders had no role in study design, data collection and analysis, decision to publish, or preparation of the manuscript."

If this statement is not correct you must amend it as needed.

Please include this amended Role of Funder statement in your cover letter; we will change the online submission form on your behalf.

►We have made the requested change to the financial disclosure in the cover letter.

3. Thank you for stating the following in the Acknowledgments Section of your manuscript:

“This research was supported by a collaborative research grant from the Virginia Tech Libraries.”

We note that you have provided funding information that is currently declared in your Funding Statement. However, funding information should not appear in the Acknowledgments section or other areas of your manuscript. We will only publish funding information present in the Funding Statement section of the online submission form.

Please remove any funding-related text from the manuscript and let us know how you would like to update your Funding Statement. Currently, your Funding Statement reads as follows:

“This research was funded, in part, by a collaborative grant from the Virginia Tech Libraries.”

Please include your amended statements within your cover letter; we will change the online submission form on your behalf.

►We have removed funding information from the Acknowledgements section. We also indicated in the cover letter a change to the funding statement.

4. When completing the data availability statement of the submission form, you indicated that you will make your data available on acceptance. We strongly recommend all authors decide on a data sharing plan before acceptance, as the process can be lengthy and hold up publication timelines. Please note that, though access restrictions are acceptable now, your entire data will need to be made freely accessible if your manuscript is accepted for publication. This policy applies to all data except where public deposition would breach compliance with the protocol approved by your research ethics board. If you are unable to adhere to our open data policy, please kindly revise your statement to explain your reasoning and we will seek the editor's input on an exemption. Please be assured that, once you have provided your new statement, the assessment of your exemption will not hold up the peer review process.

►We have agreed on a data sharing plan. Data will be made available on OSF.

5. We note that Supplementary figure in your submission contain copyrighted images. All PLOS content is published under the Creative Commons Attribution License (CC BY 4.0), which means that the manuscript, images, and Supporting Information files will be freely available online, and any third party is permitted to access, download, copy, distribute, and use these materials in any way, even commercially, with proper attribution. For more information, see our copyright guidelines: http://journals.plos.org/plosone/s/licenses-and-copyright.

We require you to either (1) present written permission from the copyright holder to publish these figures specifically under the CC BY 4.0 license, or (2) remove the figures from your submission:

1. You may seek permission from the original copyright holder of Supplementary figure to publish the content specifically under the CC BY 4.0 license.

We recommend that you contact the original copyright holder with the Content Permission Form (http://journals.plos.org/plosone/s/file?id=7c09/content-permission-form.pdf) and the following text:

“I request permission for the open-access journal PLOS ONE to publish XXX under the Creative Commons Attribution License (CCAL) CC BY 4.0 (http://creativecommons.org/licenses/by/4.0/). Please be aware that this license allows unrestricted use and distribution, even commercially, by third parties. Please reply and provide explicit written permission to publish XXX under a CC BY license and complete the attached form.”

Please upload the completed Content Permission Form or other proof of granted permissions as an "Other" file with your submission.

►Co-authors of the present manuscript are the copyright holders of the Supplemental figure. Nevertheless, to avoid further confusion, we have uploaded the copyright permission form with the current submission.

In the figure caption of the copyrighted figure, please include the following text: “Reprinted from [ref] under a CC BY license, with permission from [name of publisher], original copyright [original copyright year].”

►This text has been added to the figure in the current version of the supplemental digital content.

2. If you are unable to obtain permission from the original copyright holder to publish these figures under the CC BY 4.0 license or if the copyright holder’s requirements are incompatible with the CC BY 4.0 license, please either i) remove the figure or ii) supply a replacement figure that complies with the CC BY 4.0 license. Please check copyright information on all replacement figures and update the figure caption with source information. If applicable, please specify in the figure caption text when a figure is similar but not identical to the original image and is therefore for illustrative purposes only.

6. Please include captions for your Supporting Information files at the end of your manuscript, and update any in-text citations to match accordingly. Please see our Supporting Information guidelines for more information: <http://journals.plos.org/plosone/s/supporting-information>.

►Captions for the Supplemental digital content files have been added at the end of the manuscript and in-text citations have been edited accordingly as specified in the PLOS One guidelines.

7. Please review your reference list to ensure that it is complete and correct. If you have cited papers that have been retracted, please include the rationale for doing so in the manuscript text, or remove these references and replace them with relevant current references. Any changes to the reference list should be mentioned in the rebuttal letter that accompanies your revised manuscript. If you need to cite a retracted article, indicate the article’s retracted status in the References list and also include a citation and full reference for the retraction notice.

 ►We have reviewed the reference list. The only changes made were to accommodate the Reviewer’s request for additional references to be added.

Reviewers' comments:

REVIEWER 1

Thank you for your invitation to revise the article entitled:
A brief, theory-driven patient education video reduces high-risk over-the-counter nonsteroidal anti-inflammatory drug (NSAID) use.

►The authors thank Reviewer #1 for their insightful comments and recommendations.

I have few minor comments:
1- We need reference in line 85

►Line 85 in the submission proof reads:

Identification of an effective, low-burden intervention to de-implement high-risk OTC NSAID use has the potential to improve medication safety and population health outcomes.

This is an original assessment from our team based on our synthesis of the available evidence. As such, we were not able to add a reference here.

2- I suggest to add (in patients with CKD, HF, and/or HTN) in the last of the title to be; A brief, theory-driven patient education video reduces high-risk over-the-counter nonsteroidal anti-inflammatory drug (NSAID) use in patients with CKD, HF, and/or HTN.

►We have elected to retain the current title since “A brief, theory-driven patient education video reduces high-risk over-the-counter nonsteroidal anti-inflammatory drug (NSAID) use in patients with Chronic Kidney Disease, Heart Failure, and/or Hypertension” is an extremely long title. However, if the reviewer or editor feels strongly about the recommended title, we are willing to accept the change. Note that the three specific conditions are listed in the first line of the abstract.

3- In discussion chapter line 351; we need a reference.

►A reference has been added. Thank you for noting this omission.

4- Line 254; “In assessing the change in intent from pre- to post-intervention,…..” where is the same assessment for control group?

►Both groups were evaluated within the same regression. In this case, we evaluated the interaction between baseline intent to decrease regular use of OTC NSAIDs and group, with CONTROL as the reference group. We have modified language and added a clarifying statement to make this analysis easier for readers to understand.

5- In line 299; the authors say “After 4 weeks, intent to decrease use of OTC NSAIDs was similar in VIDEO and CONTROL” …how come while there wase a significant difference in intent between the two groups immediately after intervention (you say), the change in intent was not significantly different between groups after 4 weeks (you say) and there was no significant difference between pre and post 4 weeks period in both groups (you say)? It does make sense if there was a significant difference in intent between the two groups after two weeks also.

►We agree with the reviewer that there was opportunity to clarify results as they are complex to report due to multiple primary outcomes.

In sum, both VIDEO and CONTROL significantly increased intent to reduce OTC NSAID use over baseline levels both immediately post-intervention and 4-weeks post-intervention. At the immediately post-intervention time point, the effect of VIDEO was significantly greater than CONTROL although the group difference disappeared by 4 weeks post-intervention. Similarly, VIDEO and CONTROL significantly decreased self-reported OTC NSAID use

|  | **Immediate post-intervention** | **4 weeks post-intervention** |
| --- | --- | --- |
| Intent to reduce use of OTC NSAIDs | VIDEO>CONTROL  (both significantly higher than baseline) | VIDEO=CONTROL  (both significantly higher than baseline) |
| NSAIDs exposure | n/a | VIDEO=CONTROL  (both significantly lower than baseline) |

We are unable to ascertain why the group difference present immediately post-intervention was not sustained at 4 weeks based on data collected in the current study. In the discussion we speculate that additional reinforcement may be necessary following a single viewing of the video.

We are hopeful that the summary statement added to the end of the primary outcomes sub-section (in Results) and the related language added to the discussion address the Reviewer’s question.

In response to the Reviewer’s final point, we did not assess intent to reduce OTC NSAIDs use or self-reported OTC NSAIDs exposure 2 weeks post-intervention. This would be an excellent target for subsequent studies in order to gain insight on when the group difference present immediately after viewing the VIDEO intervention wanes. We have added this comment to the Discussion section.

6- In conclusion part the authors didn’t mention the comparison between the two studied groups as I think overall differences wasn’t found. The control group as the authors say “There was also no significant difference between VIDEO and CONTROL in NSAID Exposure at the 4-week Follow-Up assessment (19.67 (SD: 19.60) and 18.42 (SD: 15.72) dose-days, respectively; p = 0.48).” and also say “From pre-intervention to 4 weeks post-intervention, OTC NSAID Exposure decreased by 9.61 (SD: 14.26) dose-days in VIDEO (a 32.8% decrease) and 10.58 (SD: 16.10) dose-days in CONTROL (a 36.5% decrease), with no significant difference between groups (p = 0.46)”. this means (image of the Food and Drug Administration (FDA) Drug Facts label for OTC NSAIDs) that digested by control group is as effective as video (in the test group) in reducing NSAIDs exposure after the 4 weeks period of the study. The authors frankly mentioned that throughout the context of the article but, they should clarify that in the conclusion part.

►The authors thank this Reviewer for the important reminder to avoid overstating our findings, which may have occurred in the Conclusion in our attempt to concisely summarize findings.

A key take-away is that both the educational video and FDA Drug Facts label were effective in reducing intent to reduce OTC NSAIDs (immediately post-intervention and after 4 weeks) and in reducing self-reported OTC NSAIDs exposure after 4 weeks.

The VIDEO intervention was more effective than CONTROL for 1 of our 3 primary outcomes: intent to reduce OTC NSAIDs use immediately post-intervention. It was also more effective than CONTROL for participants whose baseline intent to change was lowest.

We have modified the Conclusion section accordingly. We have also made minor edits to the Abstract for the same reason.

7- Overall the article is good and concised.

►Thank you again for your thoughtful review.

**REVIEWER #2**

This manuscript discusses relevant issue which regulates around patients with CKD, DM and hypertension. The abstract describes the conduct of the study in detail. There are several missing points that could be added to the abstract for better clarity i.e. state briefly guidelines that highlight the avoidance of NSAIDs use among CKD patients, specifications on NSAIDs consumption (duration, how long, dose etc)/definiton of regular NSAID use. A brief sentence on possible reason as to why control group has greater decrease in exposure to NSAIDs could be added for better study summary.
►We thank Reviewer 2 for the insightful comments and recommendations.

We have attempted to revise the abstract to include further details but are admittedly constrained by the 300-word limit. Line 10 of the abstract includes the definition of “regular” use applied in this study.

The abstract states that the VIDEO group experienced a 32.8% decrease in self-reported NSAIDs exposure while the CONTROL group experienced a 36.5% decrease. With a p-value of 0.520, these values did not significantly so we consider the change similar among groups. We better clarified this statement in the abstract.

Methodology (Study design): Specific details on the date of RCT can perhaps be stated as "between January to September 2023". Similarly for the description under setting and participants, the specific date mentioned can be omitted.
►This change has been made in the resubmitted manuscript.

Line 109 to 118- The setting for data collection was not fully described. It is understood that background data is obtained from the EHR and emails were sent to patients to screen for eligible patients. What happened after that, patients come in for face to face interview/intervention? Appreciate if you could put in more details to help the reader understand the data collection processes. Even figure 1 did not detail out the study flow.
►Thank you for this recommendation to improve the clarity of our procedures. We have adapted the ***Setting and participants*** section to indicate that the screening instrument and trial itself were administered electronically. There was no face-to-face interaction between researchers and participants for this trial.

Line 138 " small effect size and statistical significance at p<0.05, assuming a 50%...."
►This change has been made in the resubmitted manuscript.

I like the way the video intervention was described. Very clear and concise.
►The first author very much appreciates this comment as the description required multiple iterations!

Line 196, under outcome measures, the calculation stated was not quite clear where the average NSAID exposure was calculated as 5.5 dose-days per week X 4 weeks for administration of 3-5 days of ibuprofen and 1-2 days of aspirin. It would be helpful if it could be described using a formula.
 ►We added a formula to better explain our procedures for calculating dose-days and appreciate the Reviewer’s recommendation to do so.

Conclusion- The conclusion should state that there was no significant difference between those who underwent video intervention vs the control group rather than hiding the actual outcome. This could be misleading. The manuscript clearly stated the limitations of the study, therefore, highlighting the actual outcome will be more appropriate. Perhaps, a brief statement on future work can be included to give a complete closure to the well-written manuscript.
►The authors wholeheartedly agree with the Reviewer’s recommendation and modified the conclusion accordingly.

Figure 1: I think the title 'General Study Design' is not suitable as the diagram is not showing study design.

►We have modified the title for this figure to read: Overview of Study Procedures
